# The impact of maternal experience of violence and common mental disorders on neonatal outcomes: a survey of adolescent mothers in Sao Paulo, Brazil

**DOI:** 10.1186/1471-2458-7-209

**Published:** 2007-08-16

**Authors:** Cleusa P Ferri, Sandro S Mitsuhiro, Marina CM Barros, Elisa Chalem, Ruth Guinsburg, Vikram Patel, Martin Prince, Ronaldo Laranjeira

**Affiliations:** 1 King's College London, Institute of Psychiatry, Health Service and Population Research, Section of Epidemiology, UK; 2 Department of Psychiatry, Federal University of Sao Paulo, Brazil; 3 Department of Pediatrics, Federal University of Sao Paulo, Brazil; 4 Department of Epidemiology & Population Health, London School of Hygiene and Tropical Medicine, London, UK

## Abstract

**Background:**

Both violence and depression during pregnancy have been linked to adverse neonatal outcomes, particularly low birth weight. The aim of this study was to investigate the independent and interactive effects of these maternal exposures upon neonatal outcomes among pregnant adolescents in a disadvantaged population from Sao Paulo, Brazil.

**Methods:**

930 consecutive pregnant teenagers, admitted for delivery were recruited. Violence was assessed using the Californian Perinatal Assessment. Mental illness was measured using the Composite International Diagnostic Interview (CIDI). Apgar scores of newborns were estimated and their weight measured.

**Results:**

21.9% of mothers reported lifetime violence (2% during pregnancy) and 24.3% had a common mental disorder in the past 12 months. The exposures were correlated and each was associated with low education. Lifetime violence was strongly associated with Common Mental Disorders. Violence during pregnancy (PR = 2.59(1.05–6.40) and threat of physical violence (PR = 1.86(1.03–3.35) and any common mental disorders (PR = 2.09 (1.21–3.63) (as well as depression, anxiety and PTSD separately) were independently associated with low birth weight.

**Conclusion:**

Efforts to improve neonatal outcomes in low income countries may be neglecting two important independent, but correlated risk factors: maternal experience of violence and common mental disorder.

## Background

Both the experience of violence and antenatal depression may be risk factors for adverse neonatal outcomes, particularly low birth weight. A review of studies conducted in the USA and Europe[1] showed a weak, but significant, association between abuse during pregnancy (physical, sexual or emotional) and low birth weight (pooled OR = 1.4, 95%CI = 1.1–1.8) Developing country studies are rarer, but with results in the same direction [2-4]. For antenatal mental disorder, the pattern of findings suggests that socio-economic status may be an effect modifier, with associations with low birth weight only being apparent in more deprived communities [5-9]. Although previous studies have looked at the effect of these exposures separately, they are, in fact, closely related. In a meta analysis the weighted odds ratios for the association of different mental disorders with violence among women varied from 3.5 to 5.6 [10]. The aim of this study is to describe, among disadvantaged adolescent Brazilian mothers, the association between these two exposures and their independent and interactive effects on newborn outcomes: low birth weight, small for gestational age, preterm birth, still-birth, and Apgar scores.

## Methods

### Sample and setting

The study was carried out in the Hospital Maternidade Mario de Moraes Altenfelder, the only public hospital providing obstetric care to people living in a poor neighbourhood in the north of São Paulo. Consecutive adolescents (11 to 19 years old) admitted to the hospital for obstetric care between 24/7/2001 and 27/11/2002 were invited to participate.

### Measurements

Data were collected through interviews in hospital after the women had recovered from labor and the effects of anaesthesia. This period varied between 4 to 48 hours after delivery.

#### Experience of violence

Violence was assessed using the relevant section from the Californian Perinatal Assessment[11,12]. The questions, which were translated to Portuguese and back-translated to English to ensure semantic and content validity, are:

1. Sometimes women (girls) are physically attacked by another person. Have you ever been attacked with a gun, knife or other weapon, either by a family member or a lover or friend, or by a stranger? (Subsequent questions also included a reminder to consider each of these potential perpetrators)

2. Have you ever been attacked by anyone without a weapon but with the intent to seriously injure you?

3. Have you ever been threatened with the intent to seriously harm or injure you?

4. Has anyone ever made you have any kind of sex by using physical force or by threatening to harm you?

5. Did any of these incidents occur during your pregnancy?

6. Did you ever ask for police help or for a restraining order due to domestic violence?

The first two questions were combined to generate the variable 'any physical violence' and questions one to four were combined to generate the variable "any lifetime violence". Item 4 was used to define "any sexual violence". Item 5 was used to define the exposure of any type of violence experienced during pregnancy.

#### Mental health

Mental disorders were assessed using the Composite International Diagnostic Interview (CIDI 2.1 version). The interview has been validated for use in Brazil[13,14] and all interviewers attended the accredited CIDI training centre. The primary outcome was Common Mental Disorders (CMD) in the previous 12 months; defined as all those who had a diagnosis of depression, anxiety, post-traumatic stress disorder (PTSD), somatoform or dissociative disorders at any time in the past 12 months according to the Diagnostic and Statistical Manual of Mental Disorders – 4^th ^version (DSM-IV).

#### Newborn outcomes

Five outcomes were considered: still-birth; pre term birth, Small for gestagional age (SGA), low birth weight and low Apgar scores. Babies were weighed immediately after delivery by a pediatrician using a digital scale with a precision level of 10 grams. Low birth weight was defined as < 2500 grams. A low Apgar score was defined as below 7 at 5 minutes [15]. Gestational age was calculated using the date of last menstrual period reported by participants using the New Ballard method[16]. A cutoff point of less than 37 weeks of completed gestation was used to define prematurtity[17]. Small-for-gestational-age birth was defined as birth weight below the 10^th ^percentile for expected weight according to gestational age[17], using, in the absence of any Brazilian reference data, a Canadian [18] population based gender-specific reference for birth weight.

#### Potential confounders, mediators and covariates

We also asked about the participants' age, education, socio-economic status and living arrangements. A Brazilian classification of social-economic class [19] was used, which takes into account the head of the household's education and the number of domestic electric tools in the household. It classifies individuals into five different categories (A to E) recoded to three categories: high (A and B), middle (C) and low (D and E). Obstetric history included number of pregnancies, alcohol and tobacco intake during pregnancy, pre-existing diseases (hypertension, diabetes and any 'lung, heart or kidney' diseases), and pregnancy complications (pregnancy induced hypertension, early labour, placenta praevia, placental abruption).

### Ethics

Written informed consent was sought after explanations about the aims, potential risks and benefits of the research. Interviewers offered referral to the social and mental health team of the hospital or other agencies, as appropriate. The study was approved by the ethical committee of the hospital (Hospital Maternidade Mario de Moraes Altenfelder) and the ethical committee of Federal University of Sao Paulo.

### Statistical analysis

Prevalence ratios (PR) and 95% confidence intervals were calculated for associations of violence exposures with maternal and newborn health outcomes. Poisson regression with robust variance[20] was used to estimate Prevalence Ratios and to adjust for the effect of other variables; associations of maternal mental health with violence were adjusted for age and education, while associations of maternal mental health and violence with low birth weight were initially adjusted for potential confounders (age, education, baby gender, parity, pre-existing conditions and maternal mental disorders/maternal exposure to lifetime violence), and incrementally for potential mediator factors (complications during pregnancy, alcohol intake and smoking during pregnancy and number of ante-natal consultations). An interaction between violence during pregnancy and CMD in the last 12 months was tested in the final model using a likelihood-ratio test.

## Results

One thousand and two pregnant adolescents were admitted to the hospital during the study period, representing 24.4% of the 4108 women admitted for obstetric care. One thousand adolescents agreed to participate, of whom 70, admitted for miscarriage were excluded entirely from this study (n = 930). There were eight twin pregnancies, and ten still births; Apgar scores, pre term births and small-for-gestational-age births were studied only in singleton live births leaving 912 mother/child dyads for these analyses. Low birth weight was restricted to singleton live births with 37 weeks of gestational age or above leaving 795 mother/child dyads for these analyses.

A third of the mothers were 16 or younger (Table 1). Two-thirds had completed fewer than eight years of schooling and almost half had a family income less than 400 Reais (US$120). Most cohabited with a partner. The majority had not planned their pregnancy, and were having their first baby (Table 2). One in five mothers had complications during pregnancy. Drinking and smoking were relatively uncommon. Only 4.5% (n = 42) of the participants did not present antenatally, but 30% had had fewer than the recommended six antenatal consultations. One hundred and thirty one adolescents (14.2%) had pre-term babies. 40.1 percent (n = 370) had a normal vaginal delivery while 32.2% (n = 297) had a forceps delivery and 27.7% (n = 256) had a caesarean section.

**Table 1:** The association of socio-demographic characteristics with lifetime violence and Common Mental Disorder in adolescents who have delivered babies in a public hospital in Sao Paulo.

	Frequency N(%)	Lifetime violence N = 929 N(%)	Crude PR (95% CI)	Common mental disorder N = 930 N(%)	Crude PR (95% CI)
Age			P = 0.25		P = 0.69
< = 16 years	348 (37.4)	69 (19.8)	1.00	82 (23.6)	1.00
> 16 years	582 (62.6)	134 (23.1)	1.16(0.90–1.51)	144 (24.7)	1.05 (0.83–1.33)
					
Education			P = 0.003†		P = 0.051†
0–4 years	101 (10.9)	32 (31.7)	1.00	29 (28.7)	1.00
5–8 year	491 (53.0)	112 (22.8)	0.72 (0.52–1.00)	127 (25.9)	0.90 (0.64–1.27)
9 or more years	335 (36.1)	59 (17.7)	0.56 (0.39–0.81)	70 (20.9)	0.73 (0.50–1.05)
					
Family income (R$ per month)			P = 0.25†		P = 0.57†
0–400	367 (39.5)	80(21.9)	1.00	98 (26.7)	1.00
401–1000	368 (39.6)	72 (19.6)	0.89 (0.67–1.19)	93 (25.3)	0.95(0.74–1.21)
> 1000	95 (10.2)	30 (31.6)	1.44 (1.01–2.06)	23 (24.2)	0.91(0.61–1.34)
missing values	100 (10.7)	21 (21.0)	0.96 (0.63–1.47)	12 (12)	0.45 (0.26–0.78)
					
Social Class*			P = 0.98†		P = 0.25†
A and B	62 (6.7)	15 (24.2)	1.00	13 (21.0)	1.00
C	364 (39.2)	76 (20.9)	0.86(0.53–1.40)	83 (22.8)	1.09 (0.65–1.83)
D and E	501 (54.1)	110 (22.0)	0.91(0.57–1.45)	129 (25.7)	1.23 (0.74–2.03)
					
Living with a partner			P = 0.73		P = 0.35
No	354 (38.1)	75 (21.2)	1.00	92 (26.0)	1.00
Yes	576 (61.9)	128 (22.2)	1.05 (0.81–1.35)	134 (23.3)	0.89(0.71–1.13)
					
Number of people in household			P = 0.57†		P = 0.23†
0–2 people	300 (32.4)	62 (20.7)	1.00	65 (21.7)	1.00
3–4 people	297 (32.1)	65 (21.9)	1.06 (0.78–1.44)	75 (25.3)	1.16 (0.87–1.56)
5 + people	329 (35.5)	74 (22.6)	1.09 (0.81–1.47)	85 (25.8)	1.19 (0.90–1.58)

*ANEP, 1997 (based on the electrical equipment in the household and the educational level of the head of the family

† test for trend

**Table 2:** The association of other risk factors and common mental disorders in adolescents who have delivered babies in a public hospital in Sao Paulo.

	Frequency N(%)	Lifetime violence N(%) N = 929	Crude PR (95% CI)	Common mental disorder N(%) N = 930	Crude PR (95% CI)
Planned Pregnancy			P = 0.94		P = 0.57
No	759 (81.8)	165(21.7)	1.00	182 (24.0)	1.00
Yes	169 (18.2)	37(22.0)	1.01(0.74–1.39)	44 (26.0)	1.08 (0.82–1.44)
Parity			P = 0.05		P = 0.33
> 1	173 (18.6)	47(27.2)	1.00	47 (27.2)	1.00
1	756 (81.4)	155(20.5)	0.76(0.57–1.00)	179 (23.7)	0.87 (0.66–1.15)
Ante-natal care			P = 0.45†		P = 0.32†
0 consultations	42 (4.5)	6 (14.3)	1.00	6 (14.3)	1.00
1–5	271(29.1)	71(26.2)	1.83(0.85–3.95)	68 (25.1)	1.76 (0.81–3.79)
6+	554(59.6)	113(20.4)	1.43(0.67–3.06)	139 (25.1)	1.76(0.82–3.73)
missing values	63(6.8)	13(20.6)	1.44(0.59–3.50)	12(20.6)	1.44(0.60–3.50)
Pre-existing conditions			P = 0.17		P = 0.01
No	831 (89.6)	176(21.2)	1.00	192 (23.1)	1.00
Yes	96 (10.4)	26(27.1)	1.27(0.90–1.82)	33 (34.4)	1.49 (1.10–2.01)
Obstetric complications during pregnancy			P = 0.71		P = 0.71
No	731 (78.8)	162 (22.2)	1.00	180 (24.6)	1.00
Yes	197 (21.2)	41 (20.9)	0.94(0.69–1.28)	46 (23.3)	0.94 (0.71–1.26)
Gestational age			P = 0.43		P = 0.16
> 37 weeks	789 (85.8)	170 (21.5)	1.00	185 (23.4)	1.00
= < 37 weeks	131 (14.2)	32 (24.6)	1.14(0.82–1.59)	38 (29.0)	1.24 (0.92–1.66)
Alcohol intake during pregnancy			P < 0.001†		P = 0.051†
None	676 (73.3)	131(19.4)	1.00	155 (22.9)	1.00
Occasionally (less than once a month)	223 (24.2)	58 (26.0)	1.34(1.02–1.75)	62 (27.8)	1.21 (0.94–1.56)
More than once a month	23 (2.5)	11 (47.8)	2.46(1.57–3.88)	8 (34.8)	1.52 (0.85–2.70)
Tobacco during pregnancy			P = 0.15†		P < 0.001†
0	779 (83.8)	162 (20.8)	1.00	168 (21.6)	1.00
1–6 cigarettes per day	101 (10.9)	29 (28.7)	1.33(0.98–1.93)	37 (36.6)	1.70 (1.27–2.27)
7+ cigarettes per day	50 (5.4)	12 (24.0)	1.15(0.69–1.92)	21 (42.2)	1.95 (1.37–2.77)

† test for trend

### Experience of violence

203 participants (21.8%) had experienced one or more types of violence at some time in their lives. The most common category was actual physical violence, experienced by 14% (n = 130) (evenly distributed between with and without a weapon), with threats of physical violence reported by 10% (n = 95) and 5% (n = 48) reporting sexual violence. Most of the violence went unreported; only 4% of those who suffered violence with a weapon and 17% of those reporting sexual violence had sought police help. Only 2% of mothers (n = 19) experienced violence during pregnancy; 26% of these had asked for police help.

### Mental health during pregnancy

Two hundred and twenty six participants (24.3%) were diagnosed with a Common Mental Disorder in the previous 12 months. The most common diagnosis was depression (13.0%) followed by PTSD (9.8%), anxiety disorders (5.7%), somatoform disorder (1.8%) and dissociative disorder (0.3%). There was much comorbidity between depression, anxiety and PTSD: 32.0% of those with PTSD and 39.6% of those with anxiety also had depression.

### Associations of violence and CMD with potential confounders and mediators

Low education and higher alcohol intake in pregnancy were associated with lifetime violence and CMD (tables 1 and 2). Higher parity was associated with lifetime violence (table 2), and pre-existing physical conditions and smoking with CMD. There was a non-significant trend for an association between CMD and premature birth.

### The association between violence and common mental disorder

After adjusting for age and years of education, violence was strongly associated with CMD: lifetime physical violence, PR 2.17 (95%CI 1.72–2.73); lifetime threats of physical violence, PR 2.39 (95%CI 1.89–3.02); lifetime sexual violence, PR 2.60 (95%CI 2.00–3.41); and any violence during pregnancy, PR 2.29 (95%CI 1.50–3.50). We examined the association between violence and specific types of CMD. The risk for depressive disorder was higher for mothers who had experienced lifetime physical violence (PR = 1.94, 95%CI 1.33–2.81), but associations with threats of physical violence (PR = 1.43, 95%CI 0.90–2.29), sexual violence (PR = 1.45, 95%CI 0.78–2.71) and violence during pregnancy (PR = 1.99, 95%CI 0.90–4.40) were not statistically significant. Post-traumatic stress disorder (PTSD) was more strongly associated with all of the categories of violence; actual physical violence (PR = 3.71, 95%CI 2.53–5.45), threats of violence (PR = 5.09, 95%CI 3.53–7.37), sexual violence (PR = 4.82, 95%CI 3.20–7.26) and violence during pregnancy (PR = 4.31, 95% CI 2.36–7.85). Anxiety disorders were strongly associated with actual physical violence (PR = 3.01, 95%CI 1.75–5.19); threats (PR = 3.22, 95%CI 1.80–5.77) and sexual violence (PR = 2.86, 95%CI 1.37–6.04).

### Association of maternal experiences of violence and common mental disorders with newborn outcomes

Ten babies (1.1%) were still born. Only one of these mothers had experienced lifetime violence (not during the pregnancy). There was no association between CMD and still birth, crude PR = 1.33 (95%CI 0.35–5.12). Apgar scores at five minutes were low (below 7) in 146 babies (16%). Neither lifetime violence (crude PR 1.01, 95% CI 0.70–1.44, adjusted PR (*Adjusted for age, education, baby gender, parity, pre-existing conditions, gestational age, complications during pregnancy, alcohol intake and smoking during pregnancy, ante-natal consultations and CMD/lifetime violence) = 1.11, 95%CI 0.77–1.60), nor violence during pregnancy (crude PR = 1.49, 95%CI 0.62–3.56, adjusted PR* = 1.59, 95%CI 0.73–3.44), nor CMD (Crude PR = 1.06, 95%CI 0.75–1.48; adjusted PR* = 1.00, 95%CI 0.70–1.43) was associated with low Apgar scores. 14.2% (131) were born with less than 37 weeks of gestational age. After adjustment for all potential confounders and mediators (*), pre term birth was found to be associated with common mental disorders (PR = 1.45 95% CI 1.00–2.09) but not with experience of violence. 239 babies (22.6%) were small for gestational age (SGA). After controlling for all potential confounders and mediators (*) only violence during pregnancy was associated with SGA (PR = 1.81, 95% CI 1.04–3.13).

One hundred and forty one babies (15.5%) had low birth-weight (LBW). Among those over 37 weeks of gestational age (n = 795) there were 62 babies with LBW (7.8%). There was a statistically significant trend for the risk of LBW to decrease with the number of ante-natal consultations (p-value, test for trend = 0.03). LBW was also strongly associated with pregnancy complications (crude PR = 1.85, 95% CI 1.10–3.10).

Table 3 summarizes the associations of violence and CMD with low birth-weight. There was a statistically significant crude association of all types of violence with low birth weight. However, after adjustment for potential confounders, only threat of physical violence and violence during pregnancy remained associated. After adjusting also for potential mediators (gestational age, complications during pregnancy, alcohol intake and smoking during pregnancy and ante-natal consultations) both any violence during pregnancy (PR = 2.59, 95%CI 1.05–6.40) and threat of physical violence (PR = 1.86, 95% CI 1.03–3.35) were associated with low birth weight. The association between CMD and LBW (crude PR = 2.19, 95% CI = 1.36 – 3.55) remaining significant after adjustment for potential confounders (PR = 1.93, 95% CI 1.07–3.47) and potential mediators (PR = 2.09, 95%CI 1.21–3.63). When looking at specific disorders depression PR = 2.52 (95%CI 1.43–4.44), anxiety disorders PR = 2.59 (95%CI 1.30–4.81) and PTSD PR = 1.91(95%CI 1.01–3.63) were each associated with low birth weight, after adjusting for confounders and mediators.

**Table 3:** The association of lifetime violence and Common Mental disorders with low birth-weight (LBW) in adolescents who have delivered term live babies in a public hospital in Sao Paulo n = 795.

	Low BTW in mothers with the exposure N/N total(%)	Low BTW in mothers without the exposure N/N total(%)			
**Experience of Violence**			Unadjusted PR 95% CI	Adjusted PR* 95% CI	Adjusted PR** 95% CI

Physical Violence (lifetime)	13/108 (12.0)	49/679 (7.2)	1.69(0.95–3.01)	1.36(0.74–2.48)	1.28(0.69–2.36)
Threat of Physical violence (lifetime)	13/81 (16.1)	49/714 (6.9)	2.34(1.33–4.12)	1.86(1.02–3.39)	1.86(1.03–3.35)
Sexual violence (lifetime)	7/41 (17.1)	55/754 (7.3)	2.34(1.14–4.81)	1.73(0.79–3.79)	1.23(0.54–2.80)
Any violence (lifetime)	20/170 (11.8)	41/616 (6.7)	1.87(1.13–3.07)	1.55(0.93–2.59)	1.59(0.94–2.70)
Any violence (during pregnancy)	5/16 (31.2)	56/778 (7.2)	4.99 (2.45–9.79)	3.74(1.79–7.83)	2.59 (1.05–6.4)

**Common Mental Disorder**					

Depression	14/103 (13.6)	48/692 (6.9)	1.95(1.12–3.46)	1.71(0.98–2.99)	2.52 (1.43–4.44)
Anxiety	12/43 (27.9)	50/752 (6.7)	4.19(2.42–7.27)	3.37(1.82–6.23)	2.59 (1.30–4.81))
PTSD	13/77 (16.7)	49/718 (6.8)	2.47(1.41–4.35)	1.93(0.73–3.47)	1.91 (1.01–3.63)
Any common mental disorder	24/187 (13.4)	37/608 (6.1)	2.19(1.36–3.55)	1.93(1.07–3.47)	2.09 (1.21–3.63)

*Adjusted for age, education, baby gender, parity, pre-existing conditions and common mental disorders/any lifetime violence.

**all above plus gestational age, complications during pregnancy, alcohol intake and smoking during pregnancy and ante-natal consultations (potential mediators)

The effects of violence during pregnancy and CMD in the last 12 months on birth weight were additive rather than multiplicative; with no statistical interaction in the final model (p = 0.31) (interaction term PR = 0.35 (95%CI 0.11–2.00). One hundred and seventy seven adolescents had a CMD and did not suffer violence during pregnancy (adjusted PR* for association with LBW = 2.35, 95%CI 1.36–4.05), seven had only been exposed to violence during pregnancy (PR* = 1.67, 95% CI 1.40–21.16) and 10 had suffered both exposures (PR* = 4.50, 95% CI 1.56–12.92).

## Discussion

Brazilian adolescents (10–19 years old) comprise 21% of the population[21]. Adolescent pregnancies are associated with poorer perinatal outcomes including low birth weight[22]. We found that violence and common mental disorders were common and correlated exposures among adolescent mothers attending a public obstetric hospital. Violence before pregnancy was not associated with low birth weight, unless there was a sexual element. Violence during pregnancy was less common, but was robustly associated with low birth-weight. Despite the strong associations between experience of violence and common mental disorders, the effects of each upon low birth weight were largely independent. Violence in pregnancy was associated with small-for-gestational-age but not with pre-term birth, whereas CMD was associated with pre-term birth but not SGA. Neither common mental disorder, nor experience of violence seemed to be associated with other adverse neonatal and pregnancy outcomes including pregnancy complications, still birth and Apgar scores.

The main limitation of our study is the timing of the interviews with mothers. The measurement of mental health shortly after childbirth may be confounded by emotional experiences common after childbirth. Also, recall bias is a possibility when exposures are ascertained after the outcomes have occurred. Arguably, this is more likely for still birth than for the less striking outcomes of low Apgar scores and low birth weight. However, we used a structured mental health diagnostic interview, delivered by trained interviewers who ensured that interviews were only carried out after the mother had fully recovered from childbirth. This method has been used in other studies[3,23]. Antenatal interviews would have been logistically difficult because of the patchy nature of antenatal care. Furthermore, episodes of violence and mental disorder following the interview and prior to childbirth may be missed. Maternal CMD may also have biased recall of violent events in the past. This may have led to an overestimation of the association between these two exposures. However, this should not affect our main findings and makes the finding of an independent association of both exposures with LBW even more striking. Although we adjusted for most recognized correlates of poor maternal mental health, violence and low birth-weight, the possibility of residual confounding cannot be excluded. Maternal Body Mass Index (BMI), which is associated with neonatal adverse outcomes [24] might have been a mediator in our study, linking both exposures to low birth weight. Maternal BMI was not assessed, so unfortunately we could not explore this possibility. We have used a population based Canadian reference for calculating SGA [18]. Although this is not optimal, a Brazilian reference does not exist. Gestational age was calculated using the date of last menstrual period reported by participants. Again this method is not ideal, but it is widely used in developing countries as the most reliable measure of gestational age, when, as was the case in our study, ultrasound estimates are not available. On the other hand, our study had a high proportion responding, a large sample (compared to most other studies in this field), and used standardized and validated measures of the exposures and outcomes.

The 22% prevalence of lifetime physical violence in our study is consistent with other estimates; 25% in India[25], 18% in China[26], and 13.1% [3] and 33.5%[2] in two Latin American studies of pregnant women. However, the prevalence of physical or sexual violence during pregnancy in our study (2%) is amongst the lowest reported, with other studies reporting prevalences ranging from 3.5% to 20% [3,26-28]. One explanation may be that a significant proportion of our young mothers were still in the parental home, rather than on their own with a potentially violent partner. The 24% prevalence of CMD among pregnant adolescents is similar to that typically found among pregnant adults in developed countries[29] and in the one previous study from Brazil [30]. Violence and CMD were highly correlated with each other in this population. Studies from developed countries clearly demonstrated that violence during pregnancy was associated with an increased risk for maternal mental disorders[31]. Women who experienced violence were more likely also to experience a range of other gendered disadvantages and this may act to create on oppressive atmosphere which results in poor mental health [32].

Very few studies have examined the impact of violence on newborn outcomes in developing countries. Nasir *et al*'s review[33] reported three studies, and we were able to identify three more [2-4] (Jejeebhoy, 1998; Menezes, 2005; Valdez-Santiago & Sanin-Aguirre, 1996). None focused on adolescent mothers or measured maternal mental health. A study from Mexico[2] (Valdez-Santiago & Sanin-Aguirre, 1996) of 110 mothers showed a significant association of violence during pregnancy with low birth weight after adjusting for age and parity (4.0, 95% CI 1.3–1.2). Parker et al (1994)[28] found a greater risk of LBW among abused adult women compared to adolescents in a disadvantaged community in America. There is a negative report from China[26], but only violent threats were considered. A retrospective study from India, in which women recalled their last pregnancy, showed that victims of violence were significantly more likely to have experienced still birth or infant death [4], and Menezes *et al *[3] also reported an association with neonatal mortality. Recent studies of the association between antenatal common mental disorder and low birth weight are somewhat inconsistent in their findings. Reports of positive associations have tended to come from those living in conditions of absolute or relative socio-economic disadvantage. For example, Hoffman & Hatch[7] found a positive association, but only among women from a low social economic status in the USA. Two studies from South Asia[8,9] and now our current study, of a disadvantaged population in Brazil, have also reported an independent association between CMD and LBW.

What are the possible mechanisms linking violence and mental disorder with adverse obstetric outcomes? The association between violence and low birth weight has been attributed to factors such as prematurity (caused by trauma), substance abuse (such as smoking), low socioeconomic status (leading to hunger), maternal medical problems and maternal mental illness [1,31]. The same mediators might plausibly apply to the association with mental disorder. However, the associations we report were evident after adjustment for all these factors. It is possible that the associations were mediated by poor nutrition and self-care in mothers; for example, abusers limiting access to food and antenatal care[31]. There may also be more direct biological pathways; there is accumulating evidence in humans that the hypothalamopituitary axis is in overdrive in pregnant women subjected to psychosocial stress[34]. Cortisol crosses the placenta and high levels inhibit intrauterine growth[35,36]. The fact that violence in pregnancy was associated with SGA but not with preterm babies, whereas CMD was associated with preterm birth but not SGA suggests different mechanisms for the two exposures on the pathway to low birth weight.

## Conclusion

The effect of violence on young mothers who have limited personal and social resources can be devastating. Appropriate interventions are urgently required to avoid or minimize the effects of violence on the health of the mothers and the babies. The implication for clinical practice is that all adolescent mothers should be routinely screened for the experience of violence, both lifetime and during pregnancy, as well as for mental disorder. Identification of the one should alert clinical teams to the possibility of the other, given the strong correlations that we and others have reported. Exposed mothers should be treated as 'at risk', supported intensively during the pregnancy and monitored for fetal growth. Antenatal mental disorder seems particularly likely to be associated with adverse obstetric factors (drinking and smoking, poor physical health and premature birth). At the social policy level, concerted efforts are need to combat gender-based violence not only as a human rights issue but as a major risk factor for poor maternal and newborn health; health professionals must actively engage in this advocacy.

Further research, preferably utilizing longitudinal designs, is needed to tease out the causal mechanisms linking violence with maternal and newborn outcomes. Such studies should examine not only the role of physical violence but also the influence of behaviors that cause harm without the use of physical force including neglect, humiliation and non-violent coerced sexual acts. The role of protective factors, such as social support, also merits investigation.

## Competing interests

The author(s) declare that they have no competing interests.

## Authors' contributions

CPF, VP and MP participated in the study concept and design, analysis and interpretation of data, drafting the manuscript and reviewing the manuscript for important intellectual content. MB, EC, RG, RL and SM participated in the study concept and design, acquisition of data, obtaining funding and critically reviewed the paper for important intellectual content. All authors approved the version submitted.

## Pre-publication history

The pre-publication history for this paper can be accessed here:


